# Soft electrostatic trapping in nanofluidics

**DOI:** 10.1038/micronano.2017.51

**Published:** 2017-12-04

**Authors:** Michael A. Gerspach, Nassir Mojarad, Deepika Sharma, Thomas Pfohl, Yasin Ekinci

**Affiliations:** 1 Swiss Nanoscience Institute, Basel 4056, Switzerland; 2 Laboratory for Micro and Nanotechnology, Paul Scherrer Institut, Villigen 5232, Switzerland; 3 Chemistry Department, University of Basel, Basel 4056, Switzerland; 4 Nanotechnology Group, ETH Zürich, Rüschlikon 8803, Switzerland; 5 Biozentrum, University of Basel, Basel 4056, Switzerland; 6 Biomaterials Science Center, University of Basel, Allschwil 4123, Switzerland

**Keywords:** contact-free electrostatic nanoparticle trapping, PDMS nanofluidic devices, high-throughput fabrication, single nano-object manipulation, soft-lithography

## Abstract

Trapping and manipulation of nano-objects in solution are of great interest and have emerged in a plethora of fields spanning from soft condensed matter to biophysics and medical diagnostics. We report on establishing a nanofluidic system for reliable and contact-free trapping as well as manipulation of charged nano-objects using elastic polydimethylsiloxane (PDMS)-based materials. This trapping principle is based on electrostatic repulsion between charged nanofluidic walls and confined charged objects, called geometry-induced electrostatic (GIE) trapping. With gold nanoparticles as probes, we study the performance of the devices by measuring the stiffness and potential depths of the implemented traps, and compare the results with numerical simulations. When trapping 100 nm particles, we observe potential depths of up to *Q*≅24 *k*_B_*T* that provide stable trapping for many days. Taking advantage of the soft material properties of PDMS, we actively tune the trapping strength and potential depth by elastically reducing the device channel height, which boosts the potential depth up to *Q*~200 *k*_B_*T*, providing practically permanent contact-free trapping. Due to a high-throughput and low-cost fabrication process, ease of use, and excellent trapping performance, our method provides a reliable platform for research and applications in study and manipulation of single nano-objects in fluids.

## Introduction

The unique properties of the polydimethylsiloxane (PDMS) elastomer in making integrated microfluidic systems established its applications as a standard tool in a broad range of disciplines, such as disease diagnostics^1^, cell biology^2^, drug discovery^3^, and photonics^4^, to name a few. Biocompatibility, gas permeability, optically transparency, and having a low elastic modulus are its most important chemical and physical features that allow for its implementation in such diverse fields^5^. A reduction of channel dimensions and fabricating nanofluidic PDMS devices not only provides a higher efficiency and sensitivity to analytes, but could also allow for controlled processing of objects with nanometer dimensions in soft matter such as colloids, viruses and individual macromolecules. Recent efforts in developing tailored fabrication procedures led to the fabrication of nanofluidic PDMS channels that provide laminar flows without clogging or collapse^6,7^. More advanced integrated systems were also adapted to applications in, for example, protein preconcentration^8^, DNA stretching^9^, and Raman spectroscopy^10^.

However, stable trapping and manipulation of single nano-objects in PDMS devices are hindered by the lack of techniques that produce strong retraction forces acting against the driving force of randomization, the Brownian motion. On average, every single particle contains an energy of 1/2 *k*_B_*T* for each translational dimension. However, the instantaneous velocity of a particle follows a Maxwell-Boltzmann distribution^11–13^, in which the energy of the particle can temporarily considerably exceed the average value. To compensate for these high energy kicks, typically potential depths of around 10 *k*_B_*T* are required for stable trapping of an object in solution as established by Ashkin *et al.*^14,15^.

**Figure 1:**
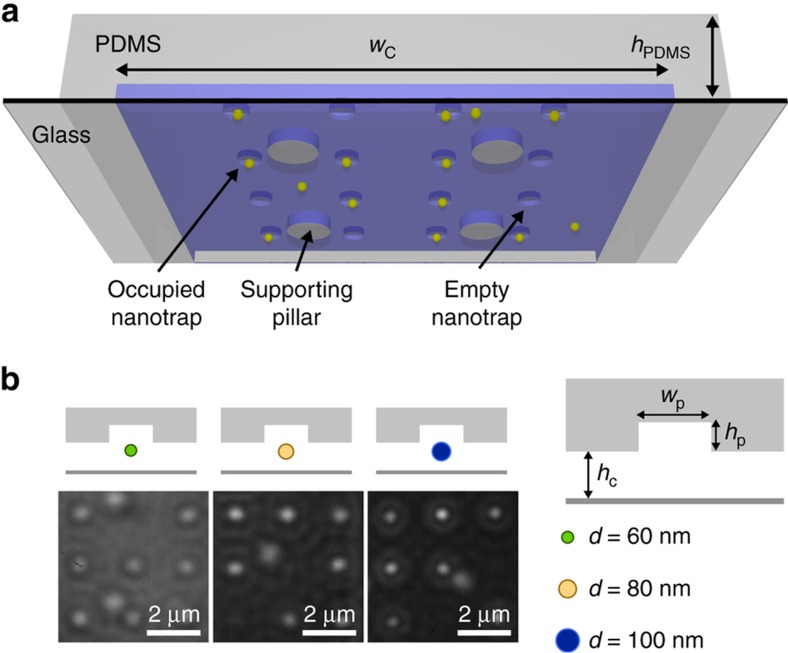
(**a**) Schematic of the polydimethylsiloxane (PDMS)-based nanofluidic trapping device with integrated pockets and supporting pillars. The nanofluidic channels had a width of *w*_c_=10 μm. Two device geometries *G*_1_ (*h*_c_=210 nm, *h*_p_=70 nm) and *G*_2_ (*h*_c_=160 nm, *h*_p_=100 nm) were used for trapping gold nanoparticles. The width *w*_p_ of the pockets varied in both device geometries from 200 to 500 nm. (**b**) Schematics and corresponding experimental optical iSCAT images of *d*=60, 80 and 100 nm gold particles trapped in circular pockets with a diameter of *w*_p_=250 nm in *G*_2_ devices. Scale bars: 2 μm.

Geometry-induced electrostatic (GIE) trapping is an effective method for parallel trapping of charged nanoparticles in a nanofluidic system^16^. This method has shown to be efficient for levitating various types of objects including metal nanoparticles of various shapes^17^, sizes^18,19^ and charges^20^, as well as polystyrene beads^16^ and lipid vesicles^16,21^. Moreover, it has been used in other applications such as single particle charge and size determination^18^, information storage^22^, and screening of electrostatic potentials^23^. The underlying physical principle of GIE-trapping is the creation of local electrostatic potential minima in a nanofluidic channel by introducing indentations in one surface of the channel. These indentations result in potential wells for stable trapping of nano-objects carrying the same sign of the net charges as that of the walls (Figure 1a). For these devices, state-of-the-art fabrication procedures play a vital role, since smallest perturbations from the ideal device geometry largely distort the potential landscape. Hence, the fabrication of GIE-trapping devices has been limited to using SiO*_x_* substrates processed by top-down nanofabrication procedures, making their production time consuming and resource demanding, practically limiting them to exploratory applications. As a result, PDMS-based GIE-trapping devices fabricated by soft lithography will substantially reduce production costs and time, making them easily to integrate into standard lab-on-a-chip systems, and making them available for research and commercial applications.

In this article, we report on the successful fabrication of PDMS-based nanofluidic systems that are used for GIE-trapping of charged nano-objects. Designing optimal device geometries, using precise fabrication techniques and an appropriate PDMS composition, preloading the device prior to bonding, and applying a tailored topographical design to avoid channel collapse play crucial roles in obtaining functional trapping devices. We use gold nanoparticles (Au NPs) down to 60 nm as probes to characterize the main trapping features such as the strength, lateral extension, and potential depth. For imaging, interferometric scattering detection (iSCAT) is used as a sensitive technique that allows for precise tracking of Au NPs as seen in Figure 1b^19,24^. Beyond passive nanoparticle trapping, we also demonstrate active manipulation of the trap stiffness by locally applying a mechanical force to elastically deform the device, a feature that is not possible in SiO*_x_*-based devices. A successful implementation of PDMS nanofluidics would allow for facile production of functional nanofluidic systems and could provide a unique platform in fields such as single-molecule force spectroscopy, pharmaceutical drug discovery, and integrated labs-on-chips.

## Materials and methods

### Device design

**Figure 2:**
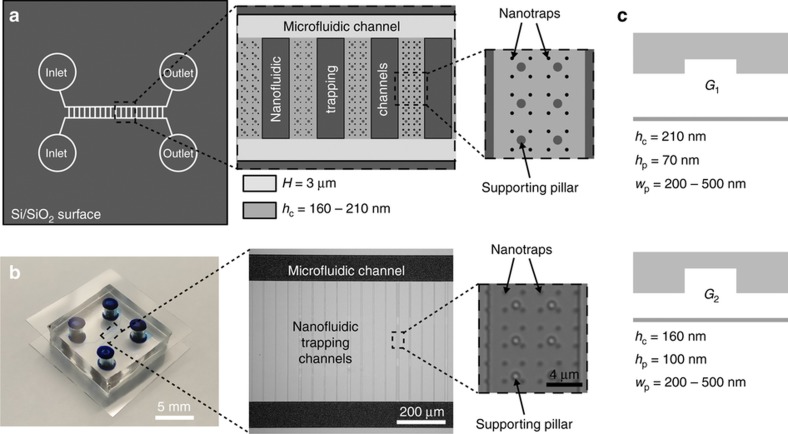
(**a**) Schematic of the silicon master design highlighting the two main features, the microfluidic reservoir channels and the nanofluidic geometry-induced electrostatic (GIE) trapping area. (**b**) Optical images of a finished polydimethylsiloxane (PDMS) device with the punched inlets and outlets and filled with a 0.2% methylene blue solution for better visualization of the microfluidic channels (left), a silicon master showing the magnified area of the microfluidic reservoir channels and the nanofluidic trapping channels (middle) and a silicon master showing one magnified nanofluidic trapping channel (right). Scale bars of images: left 5 mm, middle 200 μm, right 4 μm. (**c**) Schematic of the two device geometries used for trapping gold nanoparticles.

**Figure 3:**
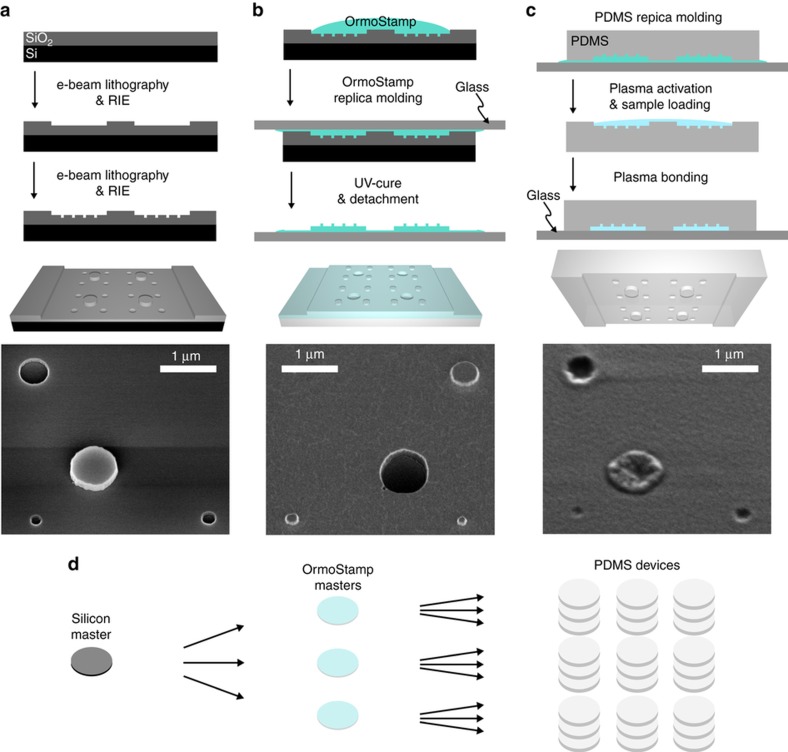
Side view sketch, three-dimensional (3D)-model and scaning electron microscope (SEM) images of the fabrication steps of the GIE trapping devices. Scale bars: 1 μm. (**a**) Fabrication of a silicon master using top-down nanofabrication tools in cleanroom facilities highlighting the main fabrication steps of the nanofluidic GIE trapping region. (**b**) Replica molding of the original silicon master using a UV curable resin (OrmoStamp) to obtain a negative master. This step can be repeated unlimited to receive multiple negative masters enabling a high-throughput production of polydimethylsiloxane (PDMS) devices. Each obtained OrmoStamp master can be repeatedly used for PDMS replica molding. (**c**) Replica molding of the negative OrmoStamp master into PDMS. The cured PDMS devices were plasma activated and covalently bound to a coverslip glass. (**d**) Sketch of high-throughput fabrication using two-step replica molding. RIE, reactive ion etching.

The design of the multi-height GIE trapping silicon master is sketched in Figure 2a. It consists of two microfluidic reservoir channels and the nanofluidic GIE trapping region. The two microfluidic channels have a depth of *H*=3 μm and a width of 100 μm, each connected by an inlet and outlet. Several GIE trapping nanofluidic channels with a length of about 0.5 mm are connected with the microfluidic channels. Within these nanofluidic channels finer nanostructures, that is, the actual nanotraps are etched. The design schematics and fabrication steps of the nanofluidic trapping region are depicted in Figures 1, 2 and 3. The nanofluidic channels have a width of *w*_c_=10 μm. Two device geometries were fabricated with a nanofluidic channel height of *h*_c_=210 nm (device geometry *G*_1_) or *h*_c_=160 nm (device geometry *G*_2_) as shown in Figure 2c. Along the width of each channel, circular indentations (pockets) with a depth of *h*_p_=70 nm (*G*_1_) or *h*_p_=100 nm (*G*_2_) and diameters of *w*_p_=200–500 nm were fabricated. Supporting pillars with a diameter of 1 μm and a spacing of 4 μm were implemented in the devices to prevent the channels from sagging and collapsing (Figures 1 and 2 and Supplementary Information S1). A final PDMS device filled with methylene blue for better visualization is shown in Figure 2b.

### Device fabrication

The transition from micro- to nanofluidics requires advanced designs, material processing and handling techniques to obtain functional PDMS devices that are micrometer in width but only nanometers in height. The fabrication steps of the nanofluidic trapping region are shown in Figure 3. A silicon master was first made (Figure 3a) by top-down fabrication methods in a cleanroom facility, namely electron beam (e-beam) lithography, followed by reactive ion etching (RIE). Next, a replica molding was carried out using a UV curable hybrid polymer (OrmoStamp, micro resist technology GmbH, 12555 Berlin, Germany) to obtain a negative copy of the original silicon master (Figure 3b)^25–27^. This step brings along two major benefits: (i) the established fabrication steps available and optimized for silicon-based GIE trapping devices do not need a re-development to obtain negative masters for making PDMS devices, and (ii) replica molding into OrmoStamp results in high-throughput fabrication because several negative OrmoStamp masters can be produced from a single silicon master, which leads to benefits in rapid replication and possibilities for commercial applications (Figure 3d). The last step, transferring the OrmoStamp into PDMS (Sylgard 184 Silicone Elastomer, Dow Corning Corporation, Midland, 48686-0994 Michigan, USA) structures (Figure 3c) was then carried out under a laminar flowbox in a conventional chemistry lab. To increase the stiffness of PDMS and thereby reduce the risk of channel collapse^28,29^, PDMS was mixed at a crosslinking rate of 5:1 (prepolymer: crosslinker) resulting in an elastic modulus of about *E*=3.6 MPa^30^. The PDMS was cured on a hotplate at 150 °C, which is the optimized temperature for high patterning resolution^31,32^ and fast crosslinking. After curing, inlet and outlet reservoirs of 4 mm diameter were punched into the PDMS device as seen in Figure 2b. The detailed nanofabrication process is provided in the Supplementary Information S2.

### Sample solution preparation

The Au NPs were purchased from BBI Solutions with a diameter *d* of 60, 80, and 100 nm (EM.GC60/80/100, BBI Solutions, CF14 5DX Cardiff, UK). 60 and 80 nm Au NPs were centrifuged two times at 2000 *g*. To exchange the buffer solution, the Au NP pellets were separated from the solution and re-suspended in fresh deionized (DI) water (18 MΩ) each time. After a third centrifugation and extraction of the excess water, a dense solution of Au NPs of ~10^11^ particles per mL was created. The extracted water was stored as a buffer solution to fill the microfluidic reservoirs and used to analyze the net charge of the particles and the ionic strength of the solution. To avoid clustering of the 100 nm Au NPs, centrifugation was done at 1500 *g* for 15 min two times and re-suspended in DI water to exchange the buffer solution. A dense solution of about 10^11^ particles per mL of 100 nm gold particles was obtained after a third centrifugation step and decantation of the supernatant. The particle zeta potentials *ζ*_p_, the solution conductivities *b*, and the diffusion coefficients *D* were measured by phase analysis light scattering (Zetasizer Nano, Malvern Instruments, WR14 1XZ Malvern, UK). For 12 different measurements of each sample, 60, 80 and 100 nm Au NPs, the measured particle zeta potentials *ζ*_p_ were −36 (±2) mV, −34 (±2) mV and −35 (±3) mV, the solution conductivities *b* were ~6.2 μS cm^−1^, ~6.1 μS cm^−1^ and ~5.5 μS cm^−1^, and the diffusion coefficients *D* were ~6.4 μm^2^ s^−1^, ~5.2 μm^2^ s^−1^ and ~4.2 μm^2^ s^−1^, respectively. From the measured zeta potentials and conductivities, the average surface charge density *σ*_p_ and particle net charges could be calculated using the semi-empirical equation^18,33^ *σ*_p_=−*ϵϵ*_0_*κ*(*k*_B_*T*/*je*)[2 sinh(*jy*/2)+(8/*κd*)tanh(*jy*/4)], with the permittivity of free space *ϵ*_0_, the dielectric constant of the medium *ϵ*, the valence of the ions *j*=1, the Debye length^34^ $\kappa^{-1}=0.304/\sqrt{c_{0}}$, and *y*=*ξ*_p_*e*/*k*_B_*T* with the elementary charge *e*. The ionic strength of the solution of 0.09 mM, 0.1 mM, and 0.1 mM for each sample of 60, 80, and 100 nm Au NPs was estimated by the linear approximation *c*_0_=1.6×10^−2^ *b*, where *c*_0_ is in units mM and *b* in μS cm^−1 ^ (Refs. 16,35). The particles net surface charge *q* for 60, 80 and 100 nm Au NPs was thus measured to be ~−92 *e*, ~−168 *e *and ~−258 *e*, corresponding to an average surface charge density of *σ*_p_~8×10^−3^ *e* nm^−2^.

### Experimental procedure

A 150-μm-thick borofloat glass microscope coverslip (Borofloat 33, Plan Optik AG, 56479 Elsoff, Germany) was rinsed with acetone, IPA and DI water and dried by nitrogen blowing. The glass and a PDMS device were air-plasma activated for 35 s at a chamber pressure of 0.5 mbar and 80% power (Femto, Diener electronic GmbH+Co. KG, 72224 Ebhausen, Germany). The PDMS parts were loaded prior bonding to the coverslip glass with a solution containing the nanoparticles to prevent collapsing during bonding^29^. Therefore, 0.25 μL of the particle solution was placed directly on the nanofluidic channel region of the PDMS. Within 1 min after the activation, the PDMS device was gently pressed to the coverslip glass. After waiting another 1 min the PDMS was covalently bound to the coverslip and could not be separated anymore. Then about 60 μL of the buffer solution was placed into each inlet, which filled the microfluidic reservoir channels by capillary forces. To stop the flow, 60 μL of the buffer solution was filled into each outlet. Finally, the device was sealed by a second cover glass to avoid evaporation of the solutions as seen in Figure 2b. The finished device was placed on the microscope holder and the particles were recorded using the iSCAT setup.

For tuning the trap stiffness, the PDMS was compressed by an applied mechanical force. In particular, a precision screw (150-801ME, Thorlabs Inc., Newton, NJ, USA) and a silicon plate of 4×4 mm^2^×0.5 mm was used. The pressure was applied to the PDMS surface by turning the screw clockwise to attain a stepwise deformation of the PDMS of about Δ*L*=15 μm each, corresponding to a pressure increase of about Δ*P*=10 kPa. The deformation pressure was calculated by taking the Young’s modulus *E*=3.6 MPa and the initial thickness of the PDMS of *h*_PDMS_=5 mm^30^.

### Electron microscopy imaging and sample preparation

To inspect if the nanostructures’ morphology is preserved during the two-step replica molding transfer into OrmoStamp and PDMS, scanning electron microscopy (SEM) images were taken. To reduce charging effects during SEM imaging, a 15 nm chromium metal layer was sputtered on the OrmoStamp and the PDMS replica mold (Leica EM SCD 500, Leica Microsystems, 35578 Wetzlar, Germany, sputtering rate 0.1 nm s^−1^). The conductive silicon master was not specially pretreated. The wafers were imaged by a Zeiss Supra 55 VP SEM (Carl Zeiss AG, Jena, Germany) using the following imaging parameters: silicon master, EHT 10 kV, InLens, WD 6 mm; OrmoStamp master, EHT 1 kV, InLens, WD 5 mm; PDMS, EHT 3 kV, SE2, WD 16 mm.

### Optical microscopy

Interferometric scattering detection (iSCAT) was used as the imaging method for particle tracking^16,24,36–39^. The iSCAT signal is generated by the interference of a reference beam, which is reflected by a strongly reflecting interface in the device, and the beam scattered from the particle^38,39^. Similar to glass-based devices^19^, PDMS systems have a weak reference beam caused by having a refractive index close to that of water, which increases the detected signal-to-noise ratio (SNR) of the trapped objects (Supplementary Information S3). The setup was built using a 300 mW diode-pumped solid-state laser (MGL-III-532, CNIlaser, Changchun, P.R. China) at *λ*=532 nm wavelength. The laser intensity was decreased and controlled using a fixed neutral density (ND) filter of OD 2 and a continuously variable ND filter wheel of OD 0–2 (NDC-50C-2, Thorlabs Inc.). An *xy* galvo deflection mirror system (GVS002, Thorlabs Inc.) was used to scan the laser over the sample running at 1 kHz rate. The laser was slightly defocused on the back focal plane of an inverted microscope (DMI 5000 M, Leica Microsystems) equipped with a ×100, 1.3 NA oil-immersion objective (HCX PL FLUOSTAR, Leica Microsystems) and an additional ×1.5 internal tube lens (11 888 699, Leica Microsystems). The reflected and scattered beams were imaged on a CMOS camera (MV-D1024-160-CL-12, Photonfocus AG, 8853 Lachen SZ, Switzerland). To synchronize the camera with the galvo deflection mirror system a four-channel AO-LabView controller (DAQ, National Instruments, Austin, TX, USA) was used and controlled by a custom made LabView software. The images for the residence time measurements were taken at an exposure time of 10 ms and an acquisition frequency from 5 to 90 Hz depending on the device geometries and particle sizes scanning a field of view 9×9 μm^2^. Images for stiffness measurements were taken at an exposure time of 1 ms and an acquisition frequency of 111 Hz scanning a field of view 1×5 μm^2^. The lateral trajectories of the particles were obtained by the center of a Gaussian profile fit to each frame, and the axial position is correlated to the amplitude of each profile fit^24,40^ (Supplementary Information S4). The average lateral localization accuracy in the *x*- or *y*-direction was δ*x*=6.5 nm for *d*=60 nm Au NPs and δ*x*=4.5 nm for *d*=80 and 100 nm Au NPs.

## Results

### Device fabrication

We used scanning electron microscopy (SEM) to determine whether the shape of the small nanostructures were preserved during the two-step replica molding transfer into OrmoStamp and PDMS. The shapes and dimensions of the 200, 250, and 500 nm pockets were well resolved in the silicon master as depicted in Figure 3a. In the 30° tilt micrograph, the height difference of the pocket depth (*h*_p_=70 nm) in comparison to the supporting pillar height (*h*_c_=210 nm) is seen. Replica molding into OrmoStamp preserved both lateral and axial dimensions of the nanometer-sized structures as well as the micrometer-wide channels and supporting pillars (Figure 3b). The flat top of the inverse pockets indicates that the resin could permeate entirely into the pockets before UV curing. To obtain functional devices, besides the device design, proper handling of the PDMS substance by controlling the mixture, curing parameters, and sample filling play critical roles (see fabrication details in Supplementary Information S2). All pocket dimensions could be transferred from OrmoStamp into PDMS as seen in the SEM image of Figure 3c. The lateral dimensions of the pockets and supporting pillars were entirely preserved during the PDMS molding. However, the well-defined axial profile of the pockets and supporting pillars in the OrmoStamp looked smoothened and more shallow in the PDMS mold.

### Electrostatic potential landscape

In GIE-trapping devices, the induced electrostatic potentials depend on a number of parameters, such as the channel and trap height, lateral trap dimension, solution ionic strength and pH, and the surface charge density of the cover glass and PDMS surfaces. Whereas SiO*_x_*-based GIE trapping devices have similar material layers on all decisive surface sides, PDMS-based GIE trapping devices consist of a top PDMS surface and a bottom glass surface layer. However, similar surface Zeta potentials of both activated PDMS and activated glass in solution of about ζ_surface_ ~−80 mV (Supplementary Information S5) indicate similar numbers of spontaneous ionization of silanol groups in water. Thus, it may be expected that the potential minimum in GIE trapping devices made from PDMS and glass substrates results in the slit midplane of the nanofluidic channel.

**Figure 4:**
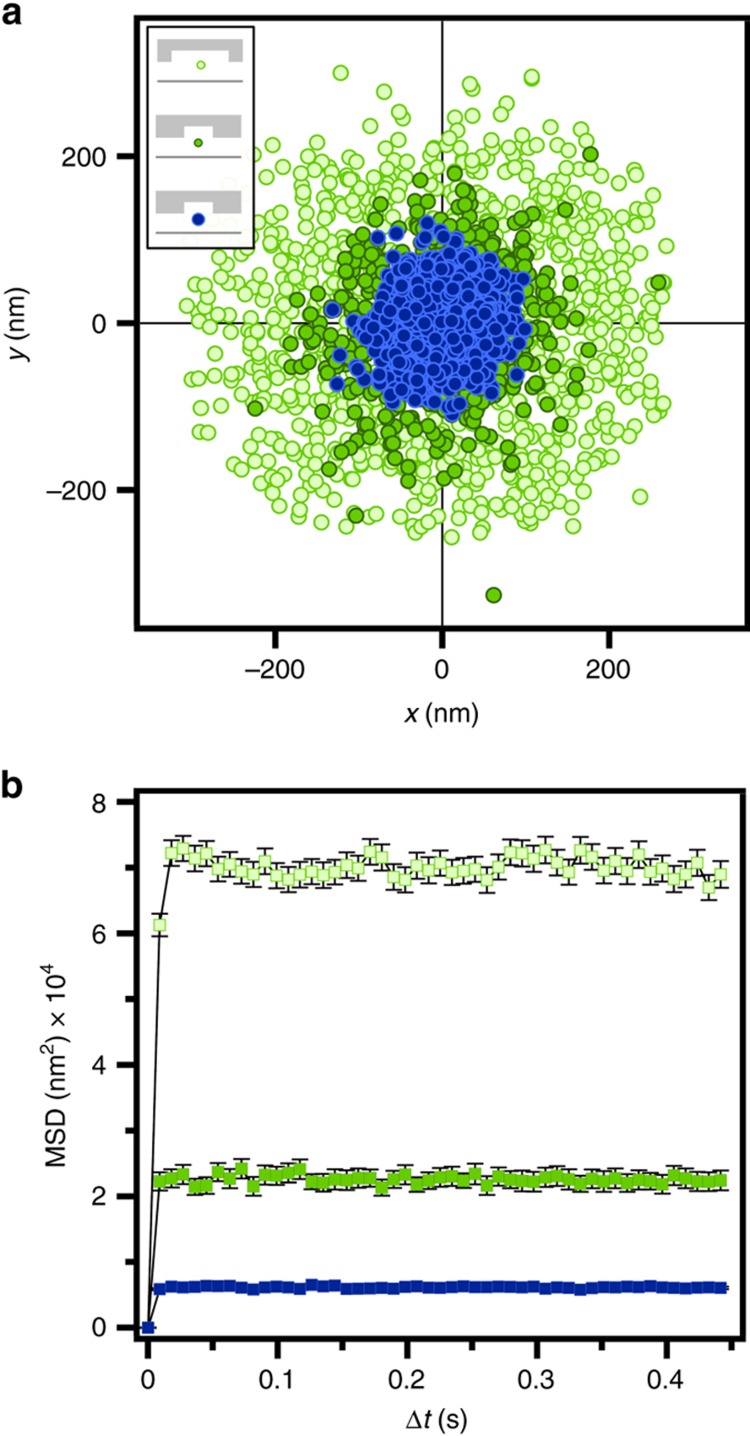
Influence of lateral trap dimensions and particle net charges on the lateral trap stiffness for devices with a channel height of *h*_c_=160 and a pocket depth of *h*_p_=100 nm (device geometry *G*_2_). (**a**) Lateral position plots of a *d*=60 nm Au NP trapped by a *w*_p_=250 nm (dark green) and a *w*_p_=500 nm (light green) circular pocket and a *d*=100 nm (blue) Au NP trapped by *w*_p_=250 nm circular pocket. (**b**) MSD plots corresponding to the lateral position plots in (**a**). The error bars denote the s.e.m. values.

The trapping strengths of different geometries were characterized by tracking the lateral motion of trapped Au NPs. Exemplary position plots of Au NPs trapped in *G*_2_ devices (*h*_c_=160 nm, *h*_p_=100 nm) with a particle diameter of *d*=60 nm in pockets of *w*_p_=250 and 500 nm and with larger particles of *d*=100 nm in *w*_p_=250 nm pockets are shown in Figure 4a. These scatterplots underline the influence of geometrical parameters and particle net charges on the spatial confinement of the particles. As expected, a smaller trap width of *w*_p_=250 nm confines the particle to smaller dimensions in comparison to larger *w*_p_=500 nm ones. Moreover, the 100 nm particles, carrying a higher net charge of ~−258 *e* compared to the ~−92 *e* of 60 nm particles, experience a stronger trapping by the pockets with the same diameter (Supplementary Movie 1). The radial symmetry of the scatterplots verifies the high replica mold fabrication quality of the lateral dimensions of the pockets as also shown from SEM inspection (Figure 3c). To quantify the trapping strength, the 2D mean-square displacement (MSD), <[Δ*r*(Δ*t*)]^2^>, was evaluated as a function of lag time Δ*t* for each series of acquired frames for a trapped particle (Figure 4b). For a particle with restricted diffusion, the MSD reaches a plateau for lag times much higher than its relaxation time *τ*_R_ in the potential well (the time a non-trapped particle would take to freely diffuse across a distance corresponding to the width of the potential well)^41^. For a harmonic potential, the plateau of the MSD is directly correlated to the radial trap stiffness *k*_r_ as^18,42^

(1)<[Δr]p2>=4kBTkr,

where *k*_B_ is the Boltzmann constant and *T* is the absolute temperature. For the 60 nm particles trapped by the *w*_p_=500 nm pockets in a *G*_2_ device, a radial trap stiffness of *k*_r_=0.22 (±0.06) fN nm^−1^ was obtained. Decreasing the trap diameter to *w*_p_=250 nm confined the motion of the 60 nm particles stronger and thus increased the trap stiffness to *k*_r_=0.8 (±0.3) fN nm^−1^, as expected. The 100 nm particles trapped by the same pockets of *w*_p_=250 nm experienced an even stronger trap stiffness of *k*_r_=2.9 (±0.9) fN nm^−1^ due to their higher net surface charge. We would like to point out that we observed no trap stiffness variation along the width of the nanofluidic channels showing that there is no roof sagging towards the middle of the channel width (Supplementary Information S1).

In addition to the lateral confinement of the electrostatic potentials, we evaluate their depth by measuring the mean residence time ${\bar{\tau }}_{K}$ (Kramers time), defined as the average time a particle dwells inside a trap before escaping. For a harmonic potential, the Kramers time is given by

(2)τ¯K≅τReQkBT,

where *k*_B_*T* is the thermal energy and Q=qΔψ the potential depth with *q* the surface net charge of the particle and Δψ the electrostatic potential difference between center of the trap and a position outside the trap in the nanofluidic channel^16,43,44^. We quantitatively analyzed the mean residence time by monitoring 100–300 escaping events for each particle size in various pocket and device geometries at a monovalent ionic concentration of *c*_0_=0.1 mM. In this procedure, ${\bar{\tau }}_{K}$ is extracted from the ‘residence time probability distribution’, *p*(*τ*), which decays exponentially with the residence time *τ* of the individual particles as^44^:

(3)p(τ)=Ae−ττ¯K.

**Figure 5:**
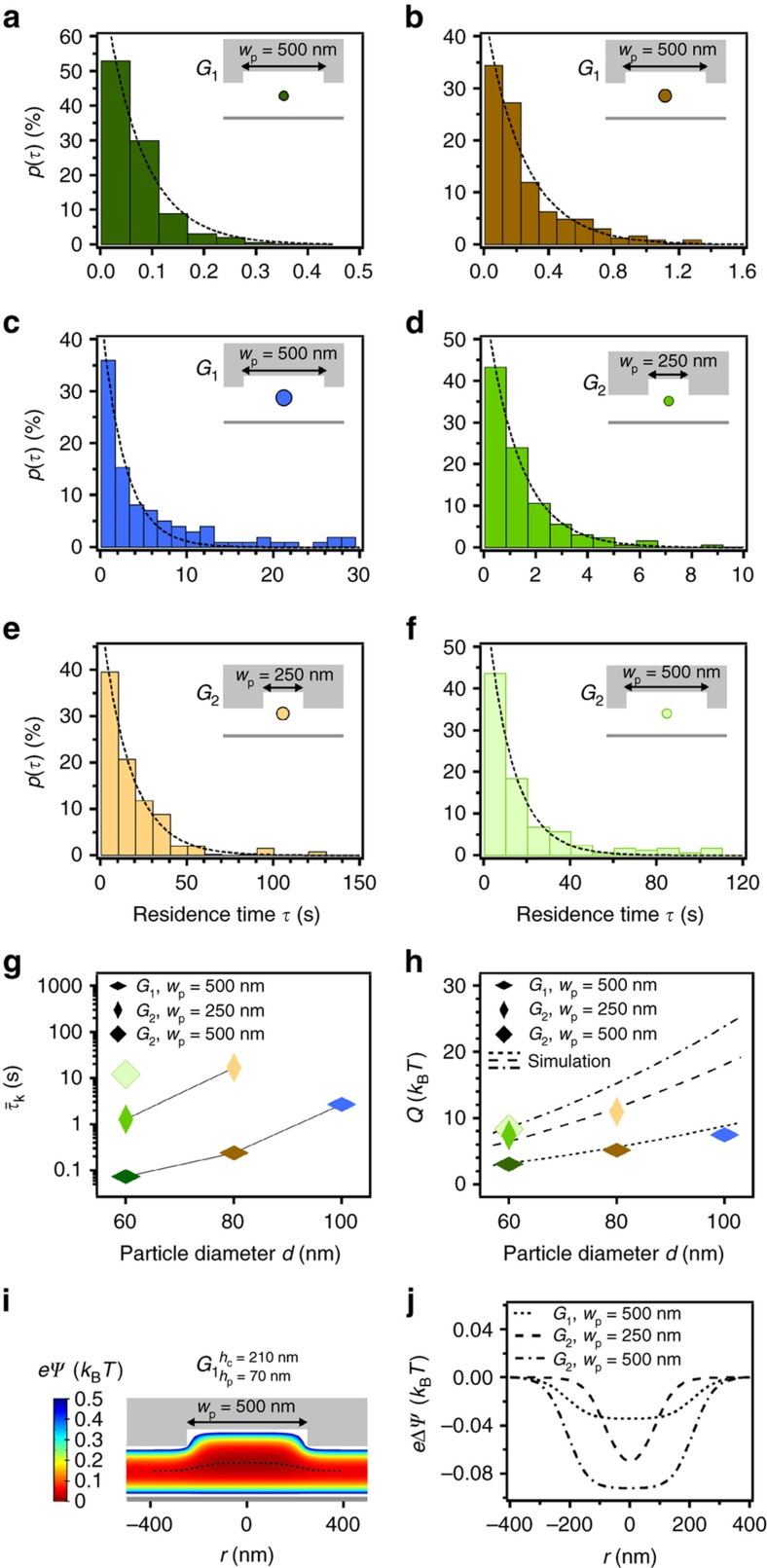
Residence time measurements. (**a**–**f**) Histograms of the residence time probability distribution showing the exponential decay of the residence times of trapped Au NPs in different device geometries (dashed lines are exponential fits) at a concentration of *c*_0_=0.1 mM. (**a**–**c**) 60 nm (dark green, **a**), 80 nm (dark brown, **b**), and 100 nm (blue, **c**) Au NPs trapped in devices with a nanofluidic channel height of *h*_c_=210 nm, a pocket depth of *h*_p_=70 nm (device geometry *G*_1_) and a width of *w*_p_=500 nm, *N*=278, 281 and 104 trapping events. (**d** and **e**) 60 nm (middle green, **d**) and 80 nm (light brown, **e**) Au NPs trapped in *G*_2_/*w*_p_=250 nm, *N*=290 and 235 trapping events. **f**) 60 nm Au NPs trapped in *G*_2_/*w*_p_=500 nm, *N*=275 trapping events. (**g**) Kramers time corresponding to the histogram distributions of (**a**–**f**) as a function of particle diameter. (**h**) Potential depths *Q* in *k*_B_*T* as a function of particle diameter calculated from the experimentally obtained Kramers time and from simulations (dashed lines). (**i**) Simulation of the electrostatic potential of a point charge of −1 *e* by solving the nonlinear Poisson-Boltzmann equation numerically for the device geometry *G*_1_ and a pocket width of *w*_p_=500 nm. (**j**) Extraction of the electrostatic potential difference of a point charge of −1 *e* for the device geometry *G*_1_/*w*_p_=500 nm, *G*_2_/*w*_p_=250 nm and *G*_2_/*w*_p_=500 nm as a function of *r* along the axial energy minimum (black dashed line in **i**).

Measured *p*(*τ*) are illustrated in Figures 5a–f and the corresponding ${\bar{\tau }}_{K}$ obtained from these graphs are plotted in Figure 5g. For different particle sizes trapped in the same geometry of *h*_c_=210 nm, *h*_p_=70 nm (*G*_1_) and with a pocket width of *w*_p_=500 nm, smaller particles escape faster from the potential wells. Sixty nanometers Au NPs carrying a net charge of ~−92 *e* quickly escaped from the traps with a very short Kramers time of ${\bar{\tau }}_{K}$=0.073 (±0.012) s. By increasing the diameter of the particles to 80 nm, carrying a higher net charge of ~−168 *e*, the Kramers time was increased to ${\bar{\tau }}_{K}$=0.242 (±0.037) s and further to ${\bar{\tau }}_{K}$=2.70 (±0.36) s for 100 nm gold particles with a net charge of ~−258 *e*. Experimentally, we find an over-exponential increase of the Kramers time as a function of particle diameter. To obtain the potential depths

(4)Q≅ln(τ¯KτR)kBT

of each system, the relaxation time *τ*_R_ was determined by the experimentally measured trap stiffness *k*_r_ and the Diffusion coefficients *D* of the particles (Table 1). In a harmonic potential, *τ*_R_ is related to the trap stiffness *k*_r_ and the Diffusion coefficients *D* of the particles as^18,45^

(5)τR=kBTDkr,

where *D*=*k*_B_*T*/3*πηd* for a particle with diameter *d* in a solution of dynamic viscosity *η*.

For the 60 nm Au NPs trapped in the *G*_1_ devices, only a potential depth of about *Q*≅3.0 *k*_B_*T* is required to be released from the 500 nm pockets, explaining the fast escape of the particles from the trap. The potential depth is increased to *Q*≅5.2 *k*_B_*T* and *Q*≅7.5 *k*_B_*T* for the 80 and 100 nm Au NPs carrying a higher net charge. From simulating the electrostatic potential of a point charge by solving the nonlinear Poisson-Boltzmann equation numerically^16^ (COMSOL Multiphysics 4.2, see Figures 5i and j and Supplementary Information S6) we can extract the potential depths for the given geometry as a function of the particle size (dashed lines in Figure 5h). For the simulations, a mean surface charge density of the particles was taken from measurements of *σ*_p_=8×10^−3^ *e* nm^−2^ and a surface charge density of the substrate glass and PDMS were estimated from spontaneous ionization in water of about *σ*_s_~3×10^−3^ *e* nm^−2^ (Ref. 46). The experimental results and the good agreement with the simulations show that the Kramers time increases over-exponentially with the particle diameter. Taking equation (2) and equation (5), this can be confirmed by the proportionality of ${\bar{\tau }}_{K}$ as

(6)τ¯K≅kBTDkreqΔψkBT∝eαd2d,

assuming the net surface charge *q* of the particles is proportional to *d*^2^, the diffusion coefficients *D* inverse proportional to *d*, and the stiffness *k*_r_ proportional to *d*^2^ $\left(q\Delta \psi \left(r\right)=k_{r}r^{2}/2\right)$^21^. A stable trapping longer than a few seconds in geometry *G*_1_ (*h*_c_=210 nm and *w*_p_=70 nm) devices and at an ionic concentration of *c*_0_=0.1 mM was only possible for the 100 nm particles. For creating deeper potential wells, the height *h*_c_ of the nanofluidic channel and the depth *h*_p_ and width *w*_p_ of the pockets are important geometrical parameters to vary. Decreasing the channel height results in deeper potentials and thus longer trapping times of the particles as well as increasing of the potential depth.

By fabricating a second design of GIE trapping devices with a reduced nanofluidic channel height of *h*_c_=160 nm and slightly deeper pockets of *h*_p_=100 nm (device geometry *G*_2_), 60 to 100 nm particles could be stably trapped by the pockets as seen in the iSCAT images of Figure 1b. The residence time probability distribution plots for the 60 nm Au NPs trapped in pocket widths of *w*_p_=250 nm and 500 nm and for the 80 nm Au NPs trapped in a pocket width of *w*_p_=250 nm are shown in Figures 5d–f. For the 60 nm and 80 nm particles trapped in the *w*_p_=250 nm pockets, Kramer times of ${\bar{\tau }}_{K}$=1.28 (±0.05) s and ${\bar{\tau }}_{K}$=16.8 (±0.3) s were obtained with a corresponding potential depth of *Q*≅7.4 *k*_B_*T* and *Q*≅10.9 *k*_B_*T*. Trapping the 60 nm particles in *w*_p_=500 nm pockets (same pocket width as for the device geometry *G*_1_), the Kramers time was increased to ${\bar{\tau }}_{K}$=12.2 (±0.3) s resulting in a potential depth of *Q*≅8.3 *k*_B_*T*. For the same pocket width, this is a 165× increase of the Kramers time compared to the ${\bar{\tau }}_{K}$=0.073 (±0.012) s measured in the *G*_1_ devices. The experimental observations confirm that particles trapped in pockets with a larger diameter have longer trapping times caused by a deeper potential (up to a certain limit) and lower counts per time of hitting the potential boundaries, due to the longer relaxation times within the larger pockets. The quantitative analysis of the Kramers time and potential depth requires statistics of many escaping events. Since for the second device design *G*_2_, the 80 nm particles in the *w*_p_=500 nm pockets and the 100 nm particles in the *w*_p_=250 nm and 500 nm pockets were stably trapped for several minutes to days the corresponding potential depths could be obtained only by simulations. For the 100 nm particles trapped by the *w*_p_=250 nm pockets in a *G*_2_ device, a potential depth of *Q*~18 *k*_B_*T* can be estimated which corresponds to a Kramers time of about ${\bar{\tau }}_{K}$~10h. When trapped in the *w*_p_=500 nm pockets, potential depths of *Q*~24 *k*_B_*T* are calculated resulting in an extremely long Kramers time beyond experimental demands.

### Tunable trapping by elastic deformation

In contrast to rigid materials, such as silicon and glass, PDMS is an elastomer that can be compressed and bent easily^47^. This provides new advantages in addition to its low-cost fabrication as discussed in previous sections. We use this unique feature of our nanofluidic system to tune the channel heights *h*_c_ and hence the trap stiffness and potential depth. Compressing the PDMS part of the devices by using mechanical forces, modifies the nanofluidic channel height and thus allows for an additional *in situ* tuning of the trap stiffness and residence times. By applying a compression pressure on the PDMS, valves and pumps in devices have been realized by, for example, pneumatic pressure^48–50^, torque actuation from embedded screws^51^ or solenoids^52^ to open and close microfluidic channels.

**Figure 6:**
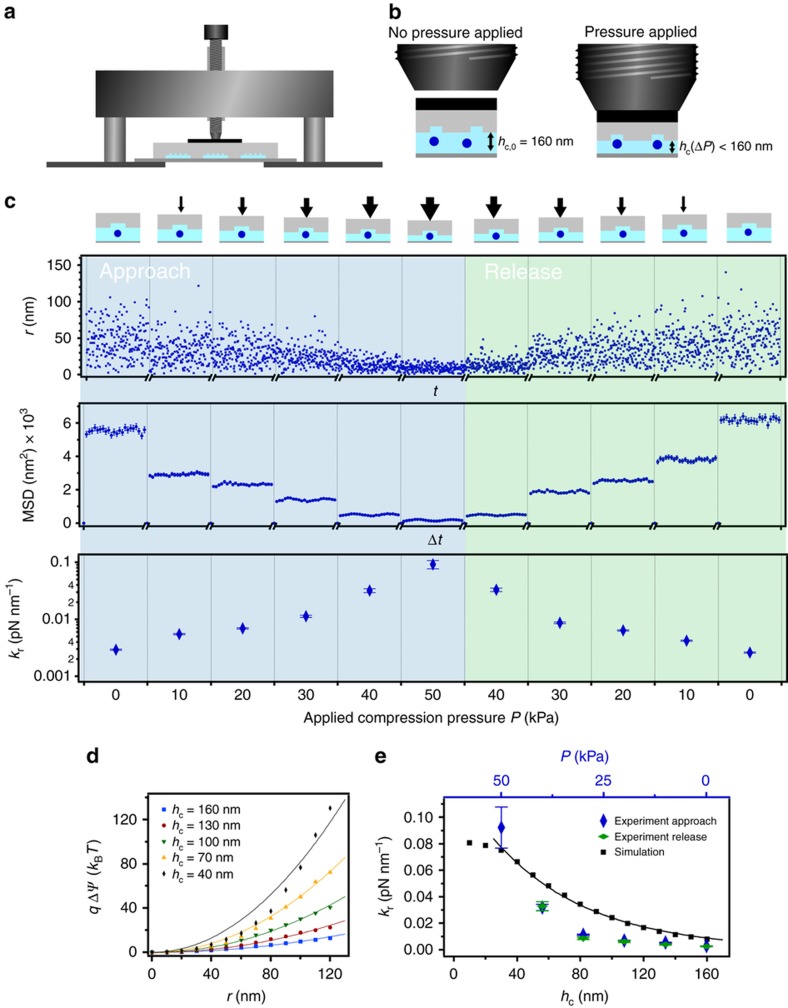
Active manipulation of trapping performance by elastic deformation of the polydimethylsiloxane (PDMS) device. (**a**) Sketch of the experimental setup using a precision screw to apply an axial compression force onto the PDMS device. (**b**) Magnified area of (**a**), illustrating the height change of the nanofluidic channel when a compression force is applied to the device. (**c**) Active manipulation of the trapping strength of a *d*=100 nm Au NP trapped in a *w*_p_=250 nm circular pocket at an initial nanofluidic channel height of *h*_c,0_=160 nm and a pocket depth of *h*_p_=100 nm. In the top graph the radial fluctuations *r* of the particle are plotted as a function of time *t* at different applied pressures. The corresponding MSDs are shown in the middle graph as a function of the lag time Δ*t*. The obtained radial trap stiffnesses from the MSDs are plotted in the lower graph as a function of the applied compression pressure *P*. Increasing the compression pressure of to the PDMS results in a nanofluidic channel height reduction and thus higher trapping strength. (**d**) Radial trap stiffness fits on simulations of the electrostatic energy along the axial energy minimum for a point charge of *q*=−258 *e* (100 nm particle) for different nanofluidic channel heights *h*_c_. (**e**) Comparison of measured and simulated values of the radial trap stiffness *k*_r_ as a function of applied compression pressure *P* and nanofluidic channel height *h*_c_.

To apply a mechanical deformation, a precision screw and a silicon plate were used as sketched in Figures 6a and b. The compression distance and the elastic modulus of the PDMS of about *E*=3.6 MPa (Ref. 30) was used to quantify the pressure exerted on the device. The result for an individual 100 nm Au NP trapped in a *w*_p_=250 nm pocket at an initial nanofluidic channel height of *h*_c,0_=160 nm and a pocket depth of *h*_p_=100 nm (device geometry *G*_2_) is shown in Figure 6c. If no compression pressure was applied on the PDMS device (*P*=0 kPa), a radial stiffness of *k*_r,0_=3×10^−3^ pN nm^−1^ was measured. Applying a stepwise deformation pressure on the PDMS device of about Δ*P* ~10 kPa each step, resulted in the reduction of the nanofluidic channel height of about Δ*h*_c_(Δ*P*) ~25 nm and thus to a stronger trap stiffness. The nanofluidic channel heights were derived by estimating the radial trap stiffness *k*_r,sim_ for different simulated channel heights according to $q\Delta \psi =k_{r,sim}r^{2}/2$ as shown in Figure 6d and comparing *k*_r,sim_ with the experimentally obtained trap stiffness (Figure 6e). Here, qΔψ represents the electrostatic energy for a point charge *q* of −258 *e*. During the approach (reduction of channel height), the particle was further laterally and axially confined, which can be seen in the decrease of the radial displacement and the corresponding MSDs in Figure 6c, the Supplementary Information S4, and the Supplementary Movie 2. At *P*=50 kPa, the radial trap stiffness increased 45× to *k*_r,50_=0.09 pN nm^−1^. At this trap stiffness, corresponding to a channel height of less than 40 nm, a potential depth of more than *Q*_max_~200 *k*_B_*T* was obtained from our simulations. Additionally, since a smaller nanofluidic channel height than the actual particle diameter was estimated, the particle could be trapped electrostatically as well as geometrically, a remarkable advantage compared to chip-based devices made from rigid materials. This process could be reversed, proving the contact-free nature of the trapping method, by releasing the pressure and going back to *P*=0 kPa, resulting in a less confined particle with the initial radial trap stiffness of about *k*_r,0_=3×10^−3^ pN nm^−1^ (Figures 6c and e).

## Discussion

Our results demonstrate that deeper potential depths as the required of 10 *k*_B_*T* for stable trapping can be obtained by GIE trapping in soft matter devices. For potential depths <10 *k*_B_*T* trapping times of only milliseconds to some seconds were observed, whereas reliable trapping of Au NPs was achieved from several seconds up to hours and days if the potential depths exceeded 10 *k*_B_*T*. Furthermore, the unique feature of soft PDMS devices to manipulate the nanofluidic channel height by an applied pressure enables straightforward tuning of the chip performance during the experiment and thus opens the capability of active trapping and releasing of nanoparticles. The performance of current chip-based GIE trapping devices made from rigid SiO_x_ materials however is characterized by their initial fabricated geometric parameters, especially the nanofluidic channel height. In addition, achieving trapping potentials of more than *Q*_max_~200 *k*_B_*T* and having the possibility of trapping the Au NPs geometrically could make the trapping of smaller nano-objects in physiological buffer conditions possible. Tuning the nanofluidic channel height during the experiments gives the possibility to load a particle solution without clogging, followed by trapping the particles by an applied pressure while still having the option to change the condition of the solution by an integrated flow fluidic system or by diffusion. Krishnan reported that even uncharged particles might be trapped in the nanofluidic indentations within potential depths >10 *k*_B_*T*, if the ratio of the particle diameter and the nanofluidic channel height *d*/*h*_c_ is larger than about 0.6 (Ref. 53). This effect was explained by the repelling of the particle from the nanofluidic channel into the trap caused by the counterions entropy of the charged channel walls. Thus, the feasibility to manipulate the nanofluidic channel heights during the experiment down to the size of the particle diameters may extend the PDMS-based trapping method for trapping even uncharged particles. For nanofluidic channel heights smaller than ~50 nm, the simulated electrostatic energies scale slightly different from the predicted correlation $q\Delta \psi =k_{r,sim}r^{2}/2$ (Figure 6d). For such small channel heights, the potential energy landscape might deviate from a perfect harmonic potential, caused by the reduced axial dimension but still constant lateral dimension of the trap width. In addition, the simulated trapping strengths deviate from our experimentally explored stiffnesses as seen in Figure 6e. We note that for the simulations no external fit parameters were used and that the simulations are based on the obtained mean values, described in section 3.2. The experiment in Figure 6 demonstrates the behavior of an individually trapped Au NP. Thus, the uncertainties in the particle size and charge and in the correlation between the applied pressure and nanofluidic channel height are the main reasons for this deviation. At small channel heights further effects such as the finite size of the particle and the effect of entropic trapping play an additional role. These effects could be studied in future experiments by exploring the variation of each effect individually.

From the viewpoint of implementation, such systems can have a great impact in the field of nanofluidics since the flexibility and low cost of fabrication bypasses the need for cleanroom facility for top-down processes. Moreover, the present method is integrated with the well-established microfluidic techniques and infrastructure, enabling the integration of GIE trapping nanofluidic devices with more complex fluidic systems such as particle sorting or trapping along concentration gradients^23^. Demonstrating that GIE trapping devices can be fabricated from replica molding processes opens additionally the possibility for fabricating such systems out of a variety of new materials. As an example, GIE trapping devices made from polystyrene foils and UV-curable adhesives could be used for X-ray scattering such as free-electron-laser studies^54,55^.

## Conclusions

We present nanofluidic trapping devices made from the elastomeric material PDMS for high-throughput fabrication and high-performance contact-free passive trapping of single charged nano-objects. These devices consist of fluidic channels that are ~160 nm in height but several micrometers in width, enabling the trapping of multiple single particles in parallel by fabricating trap lattices within the channels. Analyzing the lateral motion and residence times of the particles, we could obtain both, the trap strength and the potential depths of our traps experimentally supported by simulations. For the as-fabricated device geometries (that is, without exerted pressure), we found potentials of the electrostatic traps as deep as *Q*≅24 *k*_B_*T*, corresponding to stable trapping times of many days. We were able to actively tune the nanofluidic channel heights by applying a mechanical compression pressure and thus varying the trap stiffness and potential depths *in situ*. With this feature that is not possible in rigid SiO_x_-based devices, remarkable deep potentials of *Q*_max_~200 *k*_B_*T* and high trap stiffness of more than *k*_r,max_=0.09 pN nm^−1^ were achieved. Realizing such high potential depths could facilitate practical implementation of trapping devices for *in situ* isolation of fundamental biological entities such as macromolecules in physiological buffer conditions. Due to its ease of fabrication, our method opens the feasibility to carry out single and label-free particle research using the GIE trapping method with little effort.

## Figures and Tables

**Table 1:** Relaxation times *τ*_R_ for 60, 80, and 100 nm gold particles in different trap geometries

Device design	Device geometry *h*_c_, *h*_p_ (nm)	Trap diameter *w*_p_ (nm)	Particle diameter *d* (nm)	Diffusion coefficient *D* (μm^2^ s^−1^)	Radial stiffness *k*_r_ (fN nm^−1^)	Relaxation time *τ*_R_ (ms)
*G*_1_	210, 70	500	60	6.38	0.18	3.5
		500	80	5.23	0.55	1.4
		500	100	4.17	0.64	1.5
*G*_2_	160, 100	250	60	6.38	0.81	0.8
		250	80	5.23	2.61	0.3
		500	60	6.38	0.22	2.9

The diffusion coefficients *D* were measured using a dynamic light scattering system.
