# Supplementary material for: Soft electrostatic trapping in nanofluidics
Source: Microsyst Nanoeng. 2017 Dec 4;3:17051. doi: 10.1038/micronano.2017.51 (PMC6444982; doi:10.1038/micronano.2017.51)
Supplement: Supplementary Information [file micronano201751-s1.pdf]

Supplementary file

Soft electrostatic trapping in nanofluidics

Michael A Gerspach[1,2,3], Nassir Mojarad[4,*], Deepika Sharma[1,2,5], Thomas Pfohl[1,3,6,§] and Yasin Ekinci[1,2]

Microsystems & Nanoengineering (2017) **3,** 17051; doi:10.1038/micronano.2017.51; Published online: 4 December 2017

SAGGING OF PDMS NANOCHANNELS

A key advantage of PDMS is the possibility of actively tuning the nanofluidic channel height and therefore the trapping stiffness and potential depth by applying a compression pressure to the device. If no pressure is applied to the PDMS device, however, the design of the chip and the fabrication process including a stiffer PDMS mixture, loading of the sample solution before binding and the supporting pillars should prevent the PDMS from sagging towards the middle of the channel width (Supplementary Figure S1a). To investigate roof sagging of the PDMS nanofluidic channels, the trap stiffness at the positions 2, 4, 6 and 8 μm along the width of the channel were measured using Au NPs of $d = 100$ nm trapped in $w_p = 250$ nm pockets and at a channel height of $h_c = 160$ nm and pocket depth of $h_p = 100$ nm (device geometry G_2). In Supplementary Figure S1b the mean trap stiffness of the particles at the different positions along the nanofluidic channel is shown. Since there was no increase in the trap stiffness towards the center of the nanochannel observed and the trapping stiffnesses were constant along the channel width, roof sagging of the nanochannel along the 10 μm width can be excluded. Pure scattering standard bright field microscopy (DMI 5000 M, Leica Microsystems) equipped with a 100×, 1.3 NA oil-immersion objective (HCX PL FLUOSTAR, Leica Microsystems) and an additional 1.5× internal tube lens (11 888 699, Leica Microsystems) was used to measure multiple Au NPs at the same time with a field of view of $22 \times 22\ \mu m^2$ at an exposure time of 0.1 ms and an acquisition rate of 199 Hz using a sCMOS camera (Neo 5.5 sCMOS, Andor Technology Ltd, Belfast BT12 7AL, UK).

DEVICE FABRICATION

All features were patterned on a silicon wafer with a thermally grown SiO_2 layer of 400 nm thickness (Supplementary Figure S2a). To etch the microfluidic reservoir channels, inlets, outlets and the alignment markers, a chromium layer of 120 nm (Univex 450, Leybold GmbH, 50968 Koeln, Germany) was first evaporated, which served as a hard mask during the RIE etching of the deep channels. After spin-coating a photoresist (Microposit S1813, Dow (Shipley), Newark, DE 19713, USA; 2000 rpm, 500 rpm/s, 40 s) and UV-light exposure (Suess MA 6, Suess Microtec AG, 85748 Garching, Germany; $\lambda = 365$ nm, 120 mJ/cm^2, 12 s) using a chromium mask (Compugraphics Jena GmbH, 07751 Jena, Germany) with the design of the microfluidic system, the wafer was developed in MF-24A (Shipley, Megaposit MF-24A, Dow (Shipley)) for 40 s. The structures were etched through the chromium layer (BMP Plasmatechnology GmbH, O_2:Cl_2 with a ratio of 5:1) and further etched 3 μm into the SiO_2 (Ar 38 sccm, CHF_3 12 sccm, 100 W) and Si (SF_6 4 sccm, CHF_3 30 sccm, O_2 3 sccm, 100 W) substrate using RIE (Oxford 100, Oxford Instruments plc, Abingdon, Oxfordshire OX13 5QX, UK) as shown in Figure 2b and Supplementary Figure S2b. The remaining resist was removed in

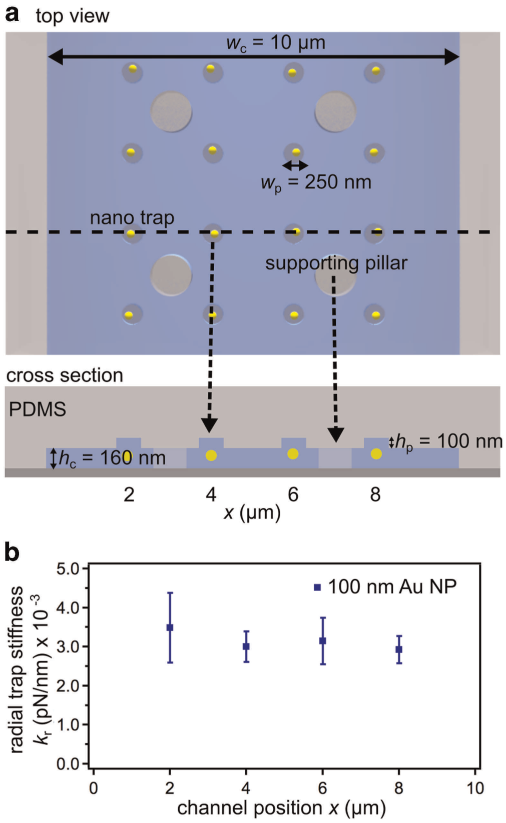

Figure S1 (**a**) Top and side view of a nanofluidic trapping channel with the integrated circular pockets and supporting pillars. The circular pockets have a distance of 2 μm from each other and are placed at the positions 2, 4, 6 and 8 μm along the width of the channel. (**b**) Mean radial trap stiffness measurements of 100 nm Au NPs trapped in $w_p = 250$ nm pockets at different positions along the width of the nanofluidic channel with a height of $h_c = 160$ nm and a pocket depth of $h_p = 100$ nm.

[1]Swiss Nanoscience Institute, Basel 4056, Switzerland; [2]Laboratory for Micro and Nanotechnology, Paul Scherrer Institut, Villigen 5232, Switzerland; [3]Chemistry Department, University of Basel, Basel 4056, Switzerland; [4]Nanotechnology Group, ETH Zürich, Rüschlikon 8803, Switzerland; [5]Biozentrum, University of Basel, Basel 4056, Switzerland and [6]Biomaterials Science Center, University of Basel, Allschwil 4123, Switzerland
Correspondence: Thomas Pfohl (thomas.pfohl69@gmail.com) and Yasin Ekinci (yasin.ekinci@psi.ch)
[*]Current address: ABB Switzerland Ltd., Semiconductors, Lenzburg 5600, Switzerland
[§]Current address: Institute of Physics, University of Freiburg, Freiburg 79104, Germany

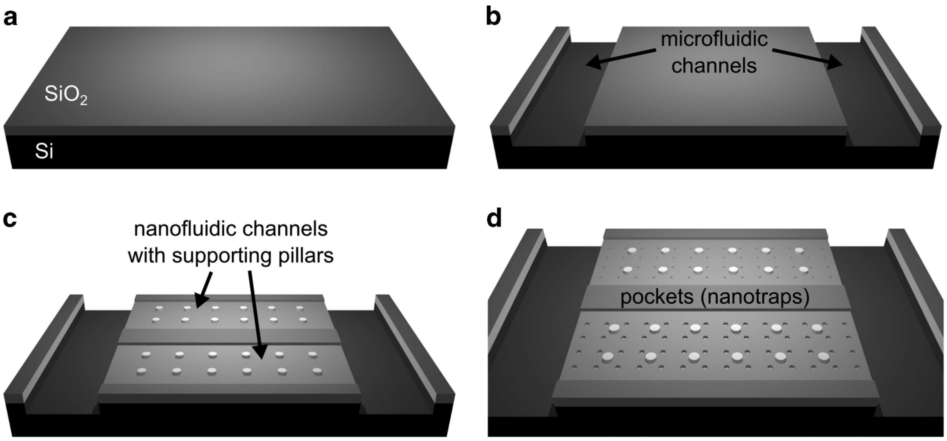

Figure S2 Schematic of the fabrication steps of the silicon master showing the microfluidic channels, nanofluidic GIE trapping region and the actual nanotraps.

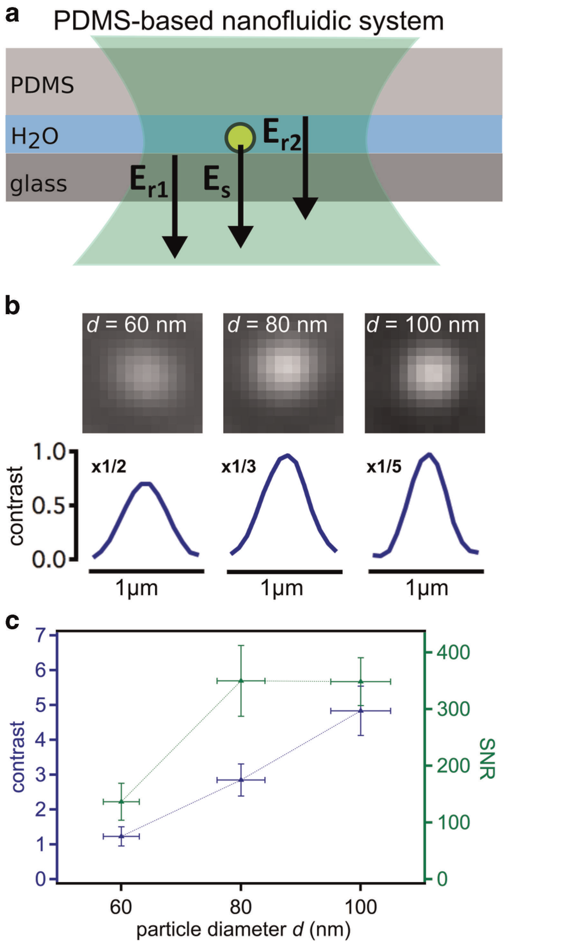

Figure S3 (**a**) Schematics of the PDMS-based nanofluidic device demonstrating the path of the incident laser beam (green) and the fields scattered from the nano-object and reflected from the glass-water and water-PDMS interfaces. Similar to glass-based nanofluidic systems, PDMS devices have a reduced reflection of the incident beam, which leads to higher SNR and contrast imaging using iSCAT. (**b**) Example of iSCAT images and the corresponding contrast profiles of 60, 80 and 100 nm gold particles as used in this paper. The contrast profiles were multiplied by 1/2, 1/3 and 1/5 for better visualization. (**c**) Mean contrast and SNR measurements as a function of particle diameter.

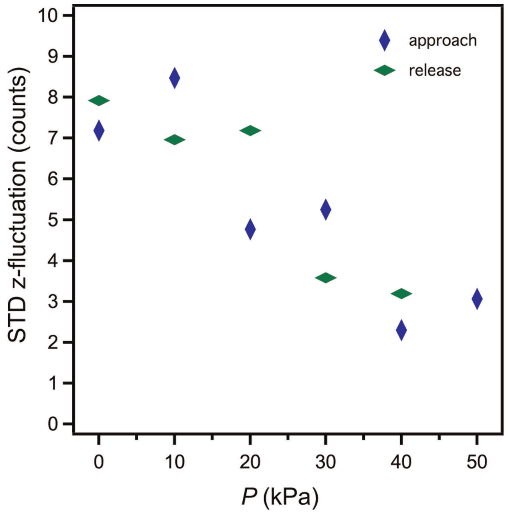

Figure S4 Standard deviation of the intensity fluctuation of the 100 nm Au NP trapped at different applied compression pressures.

acetone and the wafer was cleaned in a piranha bath (H_2SO_4(%): H_2O_2(%) = 2:1) for 20 min at 90 °C. The remaining chromium was dissolved in chromium mask etchant (Chrome ETCH No. 1, Microchemicals GmbH, 89079 Ulm, Germany) and the wafer was rigorously rinsed in DI water and dried under a nitrogen air stream. To further fabricate the nanofluidic GIE trapping region, a new chromium layer of 25 nm was evaporated on the silicon wafer. This layer was again used as a hard mask for RIE etching. After spin-coating PMMA (PMMA 950 k, Allresist GmbH, 15344 Strausberg, Germany; 4 % ethylacetate; 4000 rpm, 1000 rpm/s, 60 s) on the chromium layer, the design of parallel channels of 10 μm width and 0.5 mm length including supporting pillars were exposed using e-beam lithography (Vistec EBPG 5000 Plus, Vistec Electron Beam GmbH, 07743 Jena, Germany), developed in a mixture of methyl isobutyl ketone and isopropyl alcohol (MIBK: IPA = 1:2 (v/v), 60 s) and etched into the chromium hard mask using BMP. After removing the PMMA layer in acetone, the channels were further etched 160 nm or 210 nm deep into the silicon dioxide layer, using RIE as shown in Supplementary Figure S2c. The hard mask was removed in the chromium mask etchant. These steps were repeated to etch the actual nano-traps, circular pockets with a diameter of 200–500 nm, 70–100 nm deep into the surface of the existing channels (Supplementary

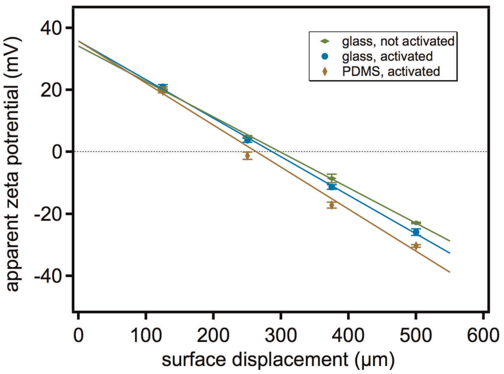

Figure S5 Surface zeta potential measurements of non-activated and activated glass and of activated PDMS in water. For activated glass and activated PDMS a similar surface zeta potential of around − 80 mV was obtained ensuring that PDMS can be used as a material for GIE trapping devices.

Figure S2d). The wafer was cleaned in a freshly made piranha solution (H_2SO_4(%):H_2O_2(%) = 2:1 (v/v)) and silanized (mixture of trichloro(1H, 1H, 2H 2H-perfluorooctyl)-silane and (tridecafluoro-1,1,2,2-tetrahydrooctyl) dimethylchlorosilane with a ratio of 1:1 (v/v)) in an evacuation chamber. The silicon wafer then served as a master to fabricate several OrmoStamp-based negative masters. Before each replica molding step from the silicon master to OrmoStamp, a new silanization of the silicon master was carried out to insure high quality OrmoStamp masters and non-sticking to the silicon wafer.

In the second main step, a cleaned 700 µm thick borofloat glass wafer (Borofloat 33, 700 µm, Schott AG, 55122 Mainz, Germany) was plasma activated for 2 min (Oxford 80, Oxford Instruments plc, O_2 20 sccm, 20 W), spincoated with an adhesion layer (OrmoPrime, micro resist technology GmbH, 4000 rpm, 45 s) for better adhesion of the OrmoStamp resin to the glass wafer and baked at 180 °C for 5 min. A 2 ml droplet of OrmoStamp hybrid polymer was placed in the middle of the silicon wafer and the glass wafer was gently aligned upside down onto the droplet and left for about 30 min until the droplet reached the edge of the two wafers. Then the silicon-OrmoStamp-glass stack was placed under a UV lamp (ELC-500, Electro-Lite Corporation, Bethel, CT 06801, USA) for 10 min to cure the hybrid polymer. After detaching the two wafers, a negative OrmoStamp-glass wafer was received. Each OrmoStamp wafer was silanized (mixture of trichloro(1H, 1H, 2H 2H-perfluorooctyl)-silane and (tridecafluoro-1,1,2,2-tetrahydrooctyl) dimethylchlorosilane with a ratio of 1:1 (v/v)) once in an evacuation chamber before the first PDMS replica molding.

The OrmoStamp wafer was used in the third main step as a negative master to obtain the PDMS-based devices. To reduce sagging and roof collapse of the thin nanometer height fluidic channels, PDMS was mixed at a ratio of 5:1 (prepolymer: crosslinker) to achieve a higher elastic modulus[1–3] of $E = 3.6$ MPa[1] and degassed in a vacuum chamber to remove air bubbles. The PDMS devices were cured on a hotplate at 150 °C for 3 h which reduced the viscosity of the PDMS prepolymer before crosslinking to achieve high resolution replication into PDMS[4,5]. The PDMS was removed from the OrmoStamp master and devices were cut out using a scalpel. Finally, inlet and outlet reservoirs of 4 mm diameter were punched into the PDMS device as seen in Figure 2b.

HIGH CONTRAST AND SNR IMAGING USING PDMS

Interferometric scattering detection (iSCAT) is used for many applications as a detection method, since it provides high sensitivity and nanometer precision detection of nano-objects down to 5 nm in diameter[6–8]. In our device configuration, the iSCAT signal is based on the interference between the scattered light from a particle and a reference beam reflected from the water-solid interface. The interference signal scales with the third power of the object diameter (d^3) whereas the pure scattering signal is proportional to d^6.

In comparison to glass-based devices[9], PDMS also has the key advantage that it is transparent from UV to IR (240–1100 nm) with a refractive index of ~ 1.4[10] in the visible range, making it possible to enclosure fluidic optical components and highly suitable for high signal-to-noise detection using iSCAT imaging. The background intensity in PDMS-based GIE trapping devices originates mainly from the reflected field of the glass-water interface E_{r1} and the water-PDMS interface E_{r2} with a reflectivity of $R_1 = 0.26\%$ and $R_2 = 0.11\%$ respectively, as sketched in Supplementary Figure S3a. Example contrast cross-section of single Au NPs with a diameter of 60, 80 and 100 nm are seen in Supplementary Figure S3b. We have analyzed over 150 frames of single Au NPs with a diameter 60, 80 and 100 nm each in nanofluidic PDMS channels. The mean contrast and SNR values are shown in the graph in Supplementary Figure S3c. We would like to point out that, using PDMS-based GIE trapping devices, the contrast and SNR values for detecting Au NPs are comparable to glass based devices and one order of magnitude higher than compared to silicon based devices[9]. This is explained due to the limited incident laser power used in silicon based devices, preventing an overexposure of the camera detector, caused by the high reflection of the Si-SiO_2 interface in the device.

REDUCTION OF AXIAL MOVEMENT OF THE PARTICLE AT REDUCED NANOFLUIDIC CHANNEL HEIGHTS

Additional to the lateral trajectories, iSCAT imaging provides information on the axial movement of the particle due to the interference signal between the scattered field of the particle and the reflected background field[8,11–13]. This information can be extracted from the intensity fluctuation of the particle and thus from the amplitude of each acquired Gaussian profile fit. For the individual trapped particle in Figure 6 of the manuscript, we obtain a decrease of the standard deviation of the intensity fluctuation of the particle for increased compression pressure (see Supplementary Figure S4). This confirms, that the reduction of the nanofluidic channel height results additionally in a stronger confinement in *z*-direction.

SURFACE ZETA POTENTIAL MEASUREMENT OF ACTIVATED GLASS AND PDMS

In glass-based GIE trapping devices, the top and bottom surface layer consist of the same material, which results in an energy minimum at the midplane of the nanofluidic channel without implemented traps. However, PDMS-based GIE trapping devices consist of a top PDMS surface and a bottom glass surface. To determine the charge properties of the glass and PDMS, surface zeta potential measurements were carried out at pH = 6.2 (Surface zeta potential cell ZEN1020, Malvern Instruments Ltd) using 1 µm polystyrene beads (micromere 01-54-103, micromod Partikeltechnologie GmbH, 18119 Rostock, Germany). The beads were diluted 1:1000 (v/v) in fresh DI water (18 MΩ). After activating a PDMS and glass sample, respectively, the apparent mobility of the tracer particles was measured at several distances away from the surfaces. Close to the surfaces, the tracer mobility is dominated by the electro-osmotic surface flow whereas far from the surface, the electrophoretic motion of the tracer particles itself dominates the mobility. By extrapolating the reported zeta potential values to zero displacement (see Supplementary Figure S5) and using the equation $\zeta_{surface} = -\zeta_{tracer}(0) + \zeta_{tracer}(\infty)$, the surface zeta potentials of the materials were obtained[14]. At pH 6.2, a zeta potential of the

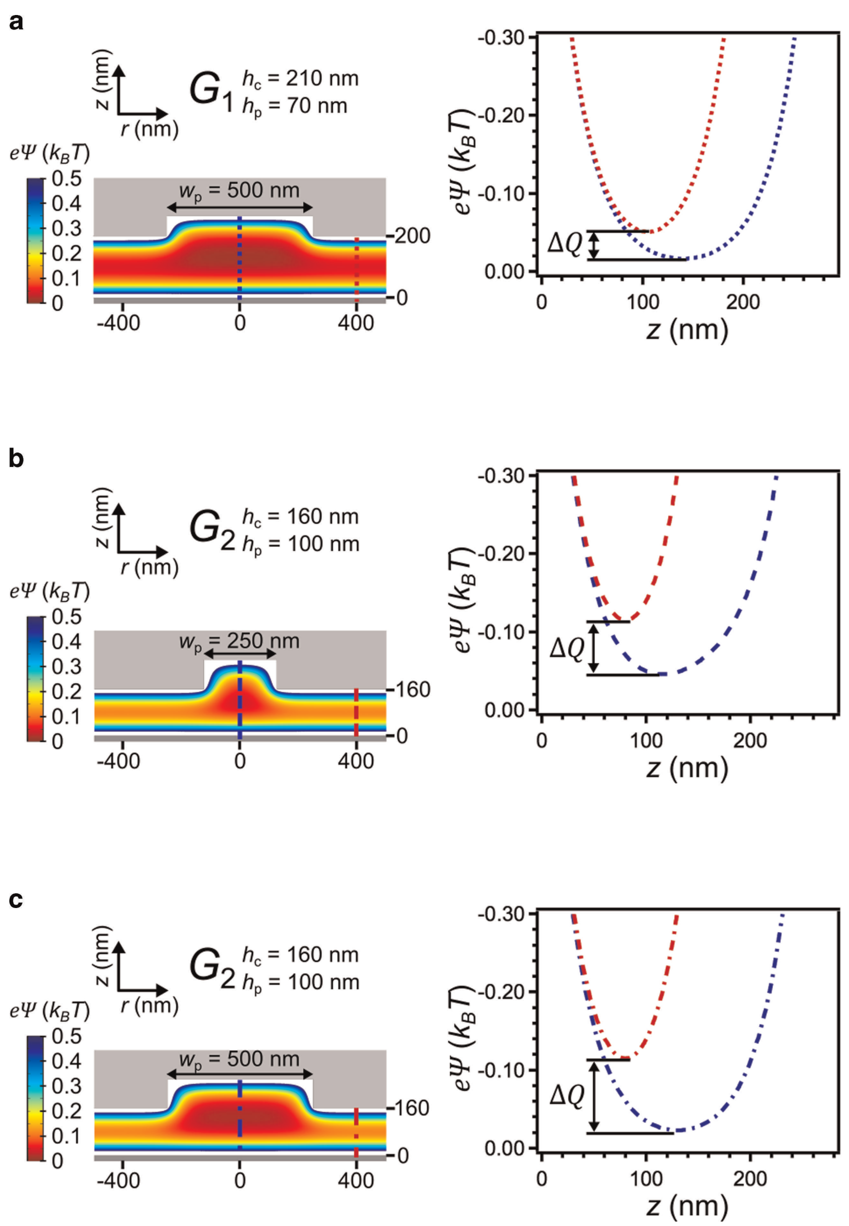

Figure S6 Two-dimensional electrostatic potentials and electrostatic energy plots along the z-axis for $r = 0$ nm and $r = 400$ nm for a point charge of -1 e for the two device geometries and pocket sizes used in the experiments.

tracer particles of $\zeta_{\text{tracer}}(\infty) = -43.8$ (±0.9) mV was measured. For activated glass and activated PDMS a surface zeta potential of $\zeta_{\text{surface,glass}} = -79.9$ (±0.9) mV and $\zeta_{\text{surface,PDMS}} = -78.2$ (±1.2) mV was obtained ensuring that the energy minimum in GIE trapping devices made from PDMS and glass as substrates results in the slit midplane of the nanofluidic channels without the trap implementations.

SIMULATION OF THE ELECTROSTATIC POTENTIALS

The simulated electrostatic potentials of a point charge of -1 e for all three device geometries (see Supplementary Figure S6) were obtained by numerically solving the nonlinear Poisson-Boltzmann equation in 3D using the COMSOL Multiphysics package 4.2 (COMSOL)[11]. An ionic strength of a monovalent ionic salt concentration of $c_0 = 0.1$ mM and a surface charge density of the Au NPs of $\sigma_p \sim 8{\cdot}10^{-3}$ e nm^{-2} were measured and taken as a boundary condition. The surface charge density of the glass and PDMS of $\sigma_s \sim 3{\cdot}10^{-3}$ e nm^{-2} were estimated to fit the simulations to the experimentally observed data in agreement with literature[15].

The circular pockets were rotationally symmetric about the $r = 0$ axis in the nanofluidic channels. The potential depths ΔQ of a point charge of -1 e were extracted by calculating the energy difference between the minimum potential along the z-axis for $r = 0$ nm (center of the pocket, blue dashed lines) and $r = 400$ nm (midplane of the nanofluidic channels outside the trap potential, red dashed lines).

REFERENCES

1 Wang Z, Volinsky AA, Gallant ND. Crosslinking effect on polydimethylsiloxane elastic modulus measured by custom-built compression instrument. *Journal of Applied Polymer Science* 2014; **41050**: 1–4.

2 Huang YY, Zhou W, Hsia KJ *et al*. Stamp collapse in soft lithography. *Langmuir* 2005; **21**: 8058–8068.

3 Lee J, Yun YK, Kim Y *et al.* PDMS nanoslits without roof collapse. *Bulletin of the Korean Chemical Society* 2009; **30**: 1793–1797.

4 Kim J-J, Lee Y, Kim HG *et al.* Biologically inspired LED lens from cuticular nanostructures of firefly lantern. *Proceedings of the National Academy of Sciences of the United States of America* 2012; **109**: 18674–18678.

5 Bender M, Plachetka U, Ran J *et al.* High resolution lithography with PDMS molds. *Journal of Vacuum Science & Technology B: Microelectronics and Nanometer Structures* 2004; **22**: 3229–3232.

6 Jacobsen V, Stoller P, Brunner C *et al.* Interferometric optical detection and tracking of very small gold nanoparticles at a water-glass interface. *Optics Express* 2006; **14**: 405–414.

7 Kukura P, Ewers H, Müller C *et al.* High-speed nanoscopic tracking of the position and orientation of a single virus. *Nature Methods* 2009; **6**: 923–927.

8 Mojarad N, Sandoghdar V, Krishnan M. Measuring three- dimensional interaction potentials using optical interference. *Optics Express* 2013; **21**: 9377–9389.

9 Gerspach MA, Mojarad N, Pfohl T *et al.* Glass-based geometry-induced electrostatic trapping devices for improved scattering contrast imaging of nano-objects. *Microelectronic Engineering* 2015; **145**: 43–48.

10 Dow Corning Corporation *Electronics Sylgard ® 184 Silicone Elastomer*. *Product Datasheet* 2013.

11 Krishnan M, Mojarad N, Kukura P *et al.* Geometry-induced electrostatic trapping of nanometric objects in a fluid. *Nature* 2010; **467**: 692–695.

12 Tae Kim J, Spindler S, Sandoghdar V. Scanning-aperture trapping and manipulation of single charged nanoparticles. *Nature Communications* 2014; **5**: 1–6.

13 Fringes S, Skaug M, Knoll AW. In situ contrast calibration to determine the height of individual diffusing nanoparticles in a tunable confinement. *Journal of Applied Physics* 2016; **119**: 1–28.

14 Malvern Instruments Surface Zeta Potential Cell (ZEN1020) 2011 1-20.

15 Behrens SH, Grier DG. The charge of glass and silica surfaces. *The Journal of Chemical Physics* 2001; **115**: 6716–6721.
